# Perplexity: evaluating transcript abundance estimation in the absence of ground truth

**DOI:** 10.1186/s13015-022-00214-y

**Published:** 2022-03-25

**Authors:** Jason Fan, Skylar Chan, Rob Patro

**Affiliations:** Center for Bioinformatics and Computational Biology, University of Maryland, College Park, USA

**Keywords:** RNA-seq, Transcript abundance estimation, Model selection

## Abstract

**Background:**

There has been rapid development of probabilistic models and inference methods for transcript abundance estimation from RNA-seq data. These models aim to accurately estimate transcript-level abundances, to account for different biases in the measurement process, and even to assess uncertainty in resulting estimates that can be propagated to subsequent analyses. The assumed accuracy of the estimates inferred by such methods underpin gene expression based analysis routinely carried out in the lab. Although hyperparameter selection is known to affect the distributions of inferred abundances (e.g. producing smooth versus sparse estimates), strategies for performing model selection in experimental data have been addressed informally at best.

**Results:**

We derive *perplexity* for evaluating abundance estimates on fragment sets directly. We adapt perplexity from the analogous metric used to evaluate language and topic models and extend the metric to carefully account for corner cases unique to RNA-seq. In experimental data, estimates with the best perplexity also best correlate with qPCR measurements. In simulated data, perplexity is well behaved and concordant with genome-wide measurements against ground truth and differential expression analysis. Furthermore, we demonstrate theoretically and experimentally that perplexity can be computed for arbitrary transcript abundance estimation models.

**Conclusions:**

Alongside the derivation and implementation of *perplexity* for transcript abundance estimation, our study is the first to make possible model selection for transcript abundance estimation on experimental data in the absence of ground truth.

## Background

Due to its accuracy, reproducibility, simplicity and low cost, RNA-seq has become one of the most popular high-throughput sequencing assays in contemporary use, and it has become the *de facto* method for the profiling of gene and transcript expression in many different biological systems. While there are many uses for RNA-seq that span the gamut from *de novo* transcriptome assembly [1, 2] through meta-transcriptome profiling [3], one of the most common uses is to interrogate the gene or isoform-level expression of known (or newly-assembled) transcripts, often with the subsequent goal of performing a differential analysis between conditions of interest.

Because of the popularity of gene and transcript expression profiling using RNA-seq, considerable effort has been expended in developing accurate, robust and efficient computational methods for inferring transcript abundance estimates from RNA-seq data. Some popular approaches focus on counting the aligned RNA-seq reads that overlap genes in different ways [4, 5]. However, these approaches have no principled way to deal with reads that align well to multiple loci (e.g. to different isoforms of a gene, or between sequence-similar regions of related genes), and this restricts their use primarily to gene-level analysis, where they may still under-perform more sophisticated approaches that attempt to resolve fragments of ambiguous origin [6].

Alternatively, many approaches offer the ability to estimate transcript-level expression using RNA-seq data (which can, if later desired by a user, be aggregated to the gene-level). The majority of these approaches perform statistical inference over a probabilistic generative model of the experiment based either on sufficient statistics of counts [7, 8] or the set of fragment alignments themselves [9]. Moreover, in addition to methods focused on deriving point estimates for transcript abundances, there has been considerable development of probabilistic Bayesian approaches for this inference problem [10–15], as well as recent attempts at multi-sample probabilistic models for simultaneous experiment-wide transcript abundance estimation [16, 17]. Bayesian approaches can sometimes offer more accurate or robust inference than methods based strictly on maximum likelihood estimation, but these Bayesian models invariably expose prior distributions, with associated hyperparameters, upon which the resulting inferences depend.

Interestingly, the recommended best practices suggested by the different Bayesian (or variational Bayesian) approaches for selecting hyperparameters differ. Specifically, Nariai et al. [12] evaluate performance varying the prior used in their variational Bayesian expectation maximization (VBEM)-based method, and they conclude that a small prior (i.e. $\alpha < 1$) leads to a sparse solution, which, in turn, results in improved accuracy. On the other hand, Hensman et al. [11] perform inference using a prior of $\alpha =1$ read per transcript. They find that, doing so, their method produces the most *robust* estimates (i.e. with the highest concordance between related replicates) that are also more accurate under different metrics that they measure. Their conclusion is that methods adopting a maximum likelihood model inferred using an expectation maximization procedure tend to produce sparse estimates close to the boundary of the parameter space which leads to less robust estimation among related samples. Unfortunately, regardless of how prior studies have argued for a “better” prior, none provide an empirical or practical procedure for model selection. Rather, they show that a value works well across a range of data under some evaluation metric, and set this as the default value for all inference tasks. Given the number of existing methods that can make use of prior information (including methods like those by Srivastava et al. [18] for single-cell data, or those by Liu et al. [19] that use orthogonal modalities of data to set priors), it becomes increasingly important to develop methods that lets one robustly and automatically select an appropriate prior (hyperparameter) for these algorithms.

To perform model (or hyperparameter) selection for transcript abundance estimators, one must be able to evaluate estimated abundances. However, evaluation of abundance estimates remains a challenge for current methods on experimental data where ground truth is completely absent. Notably, evaluation of transcript abundance estimators on experimental data have relied on careful experiment design that enables comparisons to complementary assays (e.g. correlation with qPCR) or measurements (e.g. concordance with known mixing proportions or spike-ins) [20]. Such evaluation procedures vary from study-to-study, and are simply not possible when complementary experiments are not designed or available. Thus, the natural question is then: *can the quality of transcript abundance estimates be meaningfully evaluated on the set of given fragments directly?*

It may initially be unintuitive to think that the “goodness” of a transcript abundance estimate can be evaluated in the absence of ground truth. However, in a related line of research, likelihood-based metrics for assessing the quality of *de novo* assemblies, where ground truth is unavailable, have been explored. For example, Rahman and Pachter [21] developed a method to compute the likelihoods of assembled genomes; Li et al. [22] developed a likelihood-based score to evaluate transcriptome assemblies; Smith-Unna et al. [23] developed a method to assess the quality of assembled contigs in transcriptomes; and Clark et al. [24] developed a method that is applicable to both genome and metagenomic assemblies. Furthermore, if we look to other unsupervised problem settings where ground truth annotations are absent, metrics for measuring the “goodness” of estimated models with latent parameters not only exist, but are regularly used. For example, metrics such as the silhouette score used to evaluate clustering algorithms come to mind [25]. In fact, evaluation of unsupervised probabilistic models, especially language and topic models in natural language processing, is commonplace [26, 27]. Specifically, *perplexity*, the inverse geometric mean per-*word* likelihood of a held-out test set, has been ubiquitously used to compare models [26].

In this work, we derive perplexity for transcript abundance estimation with respect to held-out per-*read* likelihoods. As we shall see, the perplexity of a held-out fragment set given an abundance estimate, computed via a quantify-then-validate approach, is a theoretically and experimentally motivated measure of the quality of the given estimate. Notably, perplexity quantifies an important biologically motivated intuition—that a good abundance estimate ought to generalize and generate the validation set, which is, in a sense, a form of a technical replicate, with high probability.

Perplexity can be used wherever the assessment of the quality of abundance estimates is desired. For example, perplexity can be used to compare different transcript abundance estimation algorithms or, as suggested above, to perform model selection to obtain the most accurate estimates from a given algorithm. In this work, we focus on experimentally assessing perplexity with respect to the latter, model selection for the prior used to estimate abundances with salmon [15]. In salmon, the reads-per-transcript prior size is a hyperparameter that controls its preference for inferring sparse or smooth abundance estimates. Notably, the problem of model selection offers a succinct assessment and immediately useful application of how perplexity can be computed to evaluate and compare the quality of candidate transcript abundance estimates.

### Contributions

Theoretically, we derive and motivate a notion of *perplexity* for transcript abundance estimation—a metric for evaluating inferred estimates in the absence of ground truth. Experimentally, we demonstrate that perplexity for transcript abundance estimates is well behaved, and establish empirical correspondence between perplexity and other metrics that are more commonly used to demonstrate the “goodness” of transcript abundance estimates.

We summarize our experimental contributions below:

1. In experimental data from the Sequencing Quality Control (SEQC) consortium [20], we show that transcript abundance estimates with the lowest perplexity (lower is better) achieve the highest correlation with complementary qPCR measurements of biological replicates.
2. In simulated data, perplexity is concordant with respect to three measurements against ground truth: Spearman correlation with respect to expressed transcripts, AUROC with respect to unexpressed transcripts, and downstream differential transcript expression analysis.
3. In a proof-of-concept style experiment, we demonstrate that perplexity can be computed for *almost any* transcript abundance estimation model.

Evidenced by these results, we propose perplexity as the first and, to our knowledge, only theoretically and experimentally justified metric for model selection for transcript abundance estimation in *experimental* data where ground truth is entirely absent.

## Preliminaries: (Approximate) Likelihood for transcript abundance estimation

Before deriving *perplexity* for transcript abundance estimation, we shall briefly recall and define the necessary objects that pertain to the *likelihood* of the probabilistic model that underpins transcript abundance estimation (as in [9, 15]).

The transcript abundance estimation problem, or quantification, from short RNA-seq *fragments* (a term used to refer, generically, to either single reads or read pairs), is the problem of assigning each fragment $f_j$ of an input fragment-set $\mathcal {F}=\{f_1,... f_N\}$ to its transcript of origin. For this work, we shall only consider quantification with respect to a given reference transcriptome whereby a quantifier maps each input fragment $f_j$ to a transcript in an input set of reference transcripts $\mathcal {T}=\{t_1,..,t_M\}$.

Given the sequence of an input fragment, said fragment may align to more than one transcript, $t_i$, in the reference transcriptome $\mathcal {T}$. Here, the *de facto* method for determining transcript of origin for fragments that multi-map to more than one transcript is to view the true fragment to transcript assignment as a latent variable, and to infer the latent variable’s expected value by performing inference in the underlying probabilistic model.

Assuming an appropriate normalization of alignment scores, we write the probability of observing a fragment, $f_j$, given that it originates from (or aligns to) transcript $t_i$ to be $P(f_j \mid t_i)$. The probability that a molecule in a sample that is selected for sequencing is the transcript $t_i$ is then $P(t_i \mid \mathbf {\theta })$, a multinomial over $\mathcal {T}$. Marginalizing over all possible alignments, the *likelihood* of observing the fragment set $\mathcal {F}$ given model parameters $\mathbf {\theta }$ is,

1 $$\begin{aligned} \mathcal {P}(\mathcal {F}\mid \mathbf {\theta }) = \prod _{j}^N \sum ^M_{i} P(t_i \mid \mathbf {\theta }) \cdot \mathcal {P}(f_j \mid t_i). \end{aligned}$$

In this work, we shall work with the *range-factorized* equivalence class approximation of the likelihood that has proven to be effective and is efficient to compute [28]. Here, sets of fragments in $\mathcal {F}$ that map to the same set of transcripts, and have similar conditional probabilities of arising from these transcripts, are said to belong to the equivalence class $\mathcal {F}^q$ (indexed by *q*). Instead of working with alignment probabilities $\mathcal {P}(f_j \mid t_i)$ of each fragment, fragments in an equivalence class $\mathcal {F}^q$ are approximated to have the same conditional probability $\mathcal {P}(f_j\mid \mathcal {F}^q, t_i)$ for mapping to each transcript $t_i$. Let ${\mathcal {C}}$ be the set of equivalence classes induced by $\mathcal {F}$ and $\Omega (\mathcal {F}^q)$ be the set of transcripts to which $f \in \mathcal {F}_q$ map. The range-factorized equivalence class approximation of the likelihood $\mathcal {P}(\mathcal {F}\mid \mathbf {\theta })$ is,

2 $$\begin{aligned} \mathcal {P}(\mathcal {F}\mid \mathbf {\theta }) \approx \prod _{\mathcal {F}^q \in \mathcal {C}} \left( {\sum _{t_i \in \Omega (\mathcal {F}^q)} P(t_i \mid \mathbf {\theta }) \cdot \mathcal {P}(f_j \mid \mathcal {F}^q, t_i)}\right) ^{N^q}. \end{aligned}$$

Here, the approximate likelihood can be computed over the number of unique equivalence classes, which is considerably smaller than the number of all possible alignments for all fragments.

## Methods

We propose a subtle but instructive change in the usual computational protocol for evaluating transcript abundance estimates. We propose a *quantify-then-validate* approach which evaluates the quality of transcript abundance estimates directly on read-sets, analogous to *train-then-test* approaches for evaluating probabilistic predictors common in natural language processing (NLP) and other fields [29, Ch. 1.3]. Instead of quantifying all available fragments and then performing evaluation with respect to complementary measurements downstream, the quantify-then-validate approach validates and evaluates the quality of a given abundance estimate directly on a set of held-out *validation* fragments withheld from inference.

We derive and adapt from NLP, the notion of *perplexity* for transcript abundance estimation for this quantify-then-validate approach [26, 27]. Perplexity is computed given only an abundance estimate, and a held-out validation set of fragments as input. Thus, perplexity evaluates the quality of abundance estimates on fragments directly and can evaluate estimates from experimental data in the absence of ground truth. Most importantly, evaluating perplexity with the quantify-then-validate approach enables quantitative, evidence-based, cross-validated selection of hyperparameters for transcript abundance estimation methods that use them.

Perplexity for transcript abundance estimation quantifies the intuition that an abundance estimate for a given sample ought, with high probability, explain and generate the set of fragments of a technical replicate. The key observation is that the likelihood $\mathcal {P}(\mathcal {F}\mid \mathbf {\theta })$ is simply a value that can be computed for any fragment set $\mathcal {F}$ and any abundance estimate $\mathbf {\theta }$ (model parameters), irrespective of whether $\mathbf {\theta }$ is inferred from $\mathcal {F}$. It is the context and application of the likelihood, $\mathcal {P}(\mathcal {F}\mid \mathbf {\theta })$, that yield semantic meaning.

Given a fragment set, ${\mathbf {F}}$, over which one seeks to infer and evaluate abundance estimates, the quantify-then-validate procedure is as follows. First, partition the input set into a *quantified* set, $\mathcal {F}$, and a *validation* set, ${\widehat{\mathcal {F}}}$. Second, *quantify* and infer abundance estimates (model parameters) $\mathbf {\theta }$ given the quantified set $\mathcal {F}$. Third, *validate* and compute the perplexity, $PP({\widehat{\mathcal {F}}}, \mathbf {\theta })$—the inverse geometric mean held-out per-read likelihood of observing the validation set, ${\widehat{\mathcal {F}}}$—given model parameters $\mathbf {\theta }$ and the validation set ${\widehat{\mathcal {F}}}$. The lower the perplexity, the better the parameters $\mathbf {\theta }$ describe the held-out fragments ${\widehat{\mathcal {F}}}$, and the better the abundance estimate parameterized by $\mathbf {\theta }$ ought to be. In fact, if we believe that the generative model is truly descriptive of the distributions that arise from the underlying biological and technical phenomena, perplexity is, in expectation, minimized when the “true” latent parameters are inferred.

Formally, given an abundance estimate $\mathbf {\theta }$, and a validation fragment-set ${\widehat{\mathcal {F}}}= \{\hat{f}_1, \dots , \hat{f}_{{\widehat{N}}}\}$, the perplexity for transcript abundance estimation is:

3 $$\begin{aligned} \begin{aligned} PP({\widehat{\mathcal {F}}}, \mathbf {\theta })&= \exp \left\{ -\frac{1}{{\widehat{N}}}\log \mathcal {P}({\widehat{\mathcal {F}}}\mid \mathbf {\theta })^{ }\right\} \\&= \exp \left\{ -\frac{1}{{\widehat{N}}}\sum ^{{\widehat{N}}}_{j=1}\log \mathcal {P}({\hat{f}}_j \mid \mathbf {\theta })^{ }\right\} , \end{aligned} \end{aligned}$$

with per-fragment likelihood,

4 $$\begin{aligned} \begin{aligned} \mathcal {P}({\hat{f}}_i \mid \mathbf {\theta }) = \sum ^M_{i=1} \mathcal {P}(t_i \mid \mathbf {\theta }) \cdot \mathcal {P}(\hat{f}_j \mid t_i). \end{aligned} \end{aligned}$$

Crucially, the probability $\mathcal {P}({\hat{f}}_j \mid \mathbf {\theta })$ of observing each held out fragment given $\mathbf {\theta }$ is computed and marginalized over the product of two terms, $\mathcal {P}({\hat{f}}_j \mid t_i)$ that depends only on the validation set of held-out fragments, and $P(t_i \mid \mathbf {\theta })$ that depends only on the given abundance estimate.

One particular application of the perplexity metric, which we explore here, is to select the best abundance estimate out of many candidate estimates arising from different hyperparameter settings for quantifiers. Thus, in this work, we use the range-factorized equivalence class approximation for perplexity (as in Eq. 2) throughout [28]. Given the range-factorized equivalence classes, ${\widehat{\mathcal {C}}}$, induced by the *validation* set, ${\widehat{\mathcal {F}}}$, (where ${\widehat{N}}^q$ is the number of fragments in an equivalence class ${\widehat{\mathcal {F}}}^q \in {\widehat{\mathcal {C}}}$) the approximation is:

5 $$\begin{aligned} PP({\widehat{\mathcal {F}}}, \mathbf {\theta }) \approx \exp \left\{ -\frac{1}{\widehat{N}} \sum _{{\widehat{\mathcal {F}}}^q \in {\widehat{\mathcal {C}}}} {\widehat{N}}^q \cdot \log \mathcal {P}({\hat{f}}_i \mid {\widehat{\mathcal {F}}}^q, \mathbf {\theta }) \right\} , \end{aligned}$$

with approximate per-fragment likelihood,

6 $$\begin{aligned} \begin{aligned} \mathcal {P}({\hat{f}}_i \mid {\widehat{\mathcal {F}}}^q, \mathbf {\theta }) = \sum _{t_i \in \Omega ({\widehat{\mathcal {F}}}^q)} P(t_i \mid \mathbf {\theta }) \cdot \mathcal {P}(\hat{f}_j \mid {\widehat{\mathcal {F}}}^q, t_i). \end{aligned} \end{aligned}$$

We use salmon’s selective-alignment based probabilistic model for conditional probabilities $\mathcal {P}({\hat{f}}_j\mid {\widehat{\mathcal {F}}}^q, t_i)$ and effective lengths of transcripts, since the model and equivalence class approximation salmon uses has proven to be a fast and effective way to approximate the full likelihood [17, 28]. For the scope of this work, salmon’s format for storing range-factorized equivalence classes conveniently contains all relevant information and values to compute perplexity with vastly smaller space requirements than would be required to store per-fragment alignment probabilities $P({\hat{f}}_j \mid t_i)$.

### “*Impossible*” fragments given parameter estimates $\mathbf {\theta }$

We now address a perplexity-related issue that is unique to evaluating transcript abundance estimates—that an observed event in the validation set may be deemed “impossible” given model parameters $\mathbf {\theta }$. The marginal probability, $\mathcal {P}({\hat{f}}_j \mid \mathbf {\theta })$, for observing a fragment ${\hat{f}}_j$ in the validation set given some abundance estimate, $\mathbf {\theta }$, may actually be zero, even if said validation fragment aligns to the reference transcriptome. This occurs exactly when all transcripts, $t_i$, to which the validation fragment ${\hat{f}}_j$ map are deemed unexpressed by $\mathbf {\theta }$ (i.e. $P(t_i \mid \mathbf {\theta }) = 0$ for all such transcripts). Here, we say that ${\hat{f}}_j$ is an *impossible fragment* given $\mathbf {\theta }$, and that $\mathbf {\theta }$ *calls* ${\hat{f}}_j$ impossible. When impossible fragments are observed in the validation set, perplexity is not a meaningful measurement.

To illustrate how impossible fragments come to be, consider the toy example in which all fragments in a quantified set that align to transcripts *A*, *B*, or *C* only ambiguously map to $\{A, B\}$, or to $\{A, C\}$. That is, no such fragments uniquely map—a phenomenon observed rather frequently for groups of similar isoforms expressed at low to moderate levels. Now, suppose that an abundance estimation model assigns all such fragments to transcript *A* and produces an estimate $\mathbf {\theta }$. The quantifier may be satisfying a prior that prefers sparsity; or prefers to do so because transcript *A* is considerably shorter than transcripts *B* and *C*, which gives it a higher conditional probability under a length normalized model. In this case, the marginal probability, $\mathcal {P}({\hat{f}}_j \mid \mathbf {\theta })$, of observing a validation fragment ${\hat{f}}_j$ that maps to $\{B,C\}$ is exactly zero given the parameters $\mathbf {\theta }$.

**Fig. 1:**
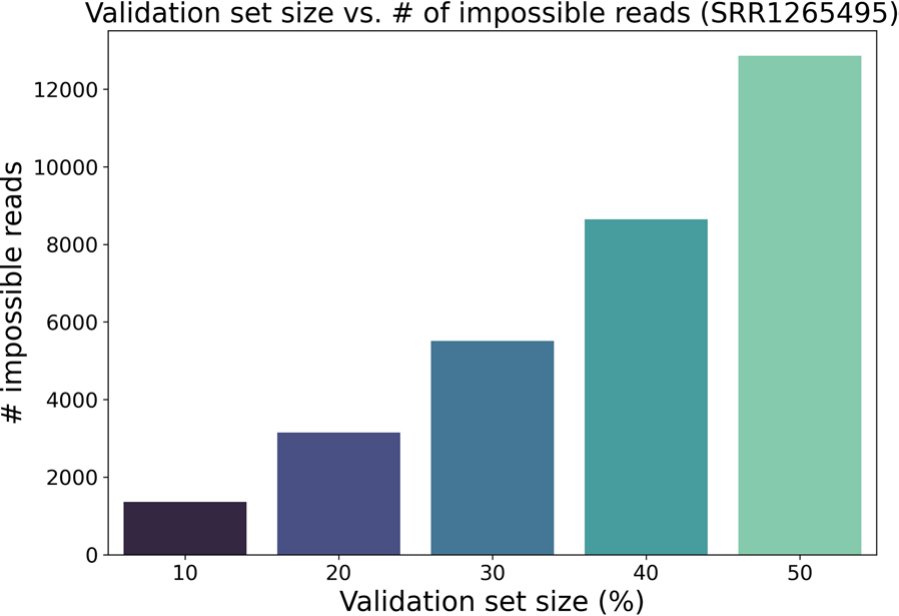
Number of fragments called impossible versus withheld validation fragment set size for sample SRR1265495. All remaining fragments are used to estimate abundances using salmon’s VBEM model using default parameters (i.e. using a prior size of 0.01 reads-per-transcript)

As an example, we randomly withhold varying percentages of fragments from one sample (SRR1265495) as validation sets and use all remaining fragments to estimate transcript abundances with salmon’s default model (i.e. the VBEM model using prior size of 0.01 reads-per-transcript). Figure 1 shows that at all partitioned percentages, impossible fragments in the validation set are prevalent with respect to estimated abundances. In fact, due to the prevalence of impossible reads, perplexity as written in Eq. 5 is undefined (or infinite) for all estimates and all validation sets in the experiments below. An important observation in both the toy and experimental examples is that there likely exist better abundance estimates that would call fewer fragments impossible, while still assigning high likelihood to the rest of the (possible) fragments. For example, an abundance estimate that reserves even some small probability mass to transcript *B* in the toy example would not call the validation fragments in question impossible.

### Why perplexities need to be *smoothed*

The problem with impossible fragments is not only that they exist. The problem is that, for a fixed validation fragment set, perplexity deems an abundance estimate that calls even one fragment impossible equally as bad as an abundance estimate that calls all fragments impossible. Here, both estimates would have unbounded perplexity since the validation set has zero likelihood given each estimate. However, the former ought be preferred over the latter.

Other fields that have adopted and used perplexity (e.g. natural language processing) usually sidestep the issue of impossible events entirely both by construction and pre-processing, working only with smoothed probabilistic models in which no event has probability zero, or removing rare words from input language corpora. However, neither strategy is available nor appropriate for evaluating transcript abundance estimates. It is neither reasonable nor useful to amend and modify each of the many modern quantifiers to produce smooth outputs (outputs in which no transcript has truly zero abundance), and fragments and transcripts cannot be pre-processed away since the set of expressed transcripts cannot be identified *a priori*. One may also be tempted to simply remove impossible fragments from a validation set, ${\widehat{\mathcal {F}}}$, before computing a perplexity or hold out fragments—but this also is not a valid strategy. This is because two different abundance estimates $\mathbf {\theta }$ and $\mathbf {\theta }^\prime$ may call different validation fragments in ${\widehat{\mathcal {F}}}$ impossible, and comparisons of likelihoods $P({\widehat{\mathcal {F}}}^\prime \mid \mathbf {\theta }^\prime )$ and $P({\widehat{\mathcal {F}}}\mid \mathbf {\theta })$ are only meaningful if the validation sets are the same (i.e. ${\widehat{\mathcal {F}}}= {\widehat{\mathcal {F}}}^\prime$). Furthermore, there is no straightforward strategy to sample and hold-out validation fragments so that no fragments are impossible. This is because most validation fragments cannot be determined to be impossible prior to abundance estimation, and any non-uniform sampling strategy would alter the underlying distributions that estimators aim to infer. To compare estimates that may call different validation fragments impossible, the proposed perplexity metric (as in Eq. 5) must be *smoothed*. Strategies that smooth perplexities ought *penalize* estimates that call fragments impossible. That is, impossible fragments under such smoothing strategies ought result in a penalty and overcome the shrinkage of $\mathcal {P}({\widehat{\mathcal {F}}}\mid \mathbf {\theta })$ to zero. Below, we detail two such smoothing strategies for computing perplexities: (a) *Laplacian smoothed perplexity* and (b) *Good-Turing smoothed* perplexity.

**Fig. 2:**
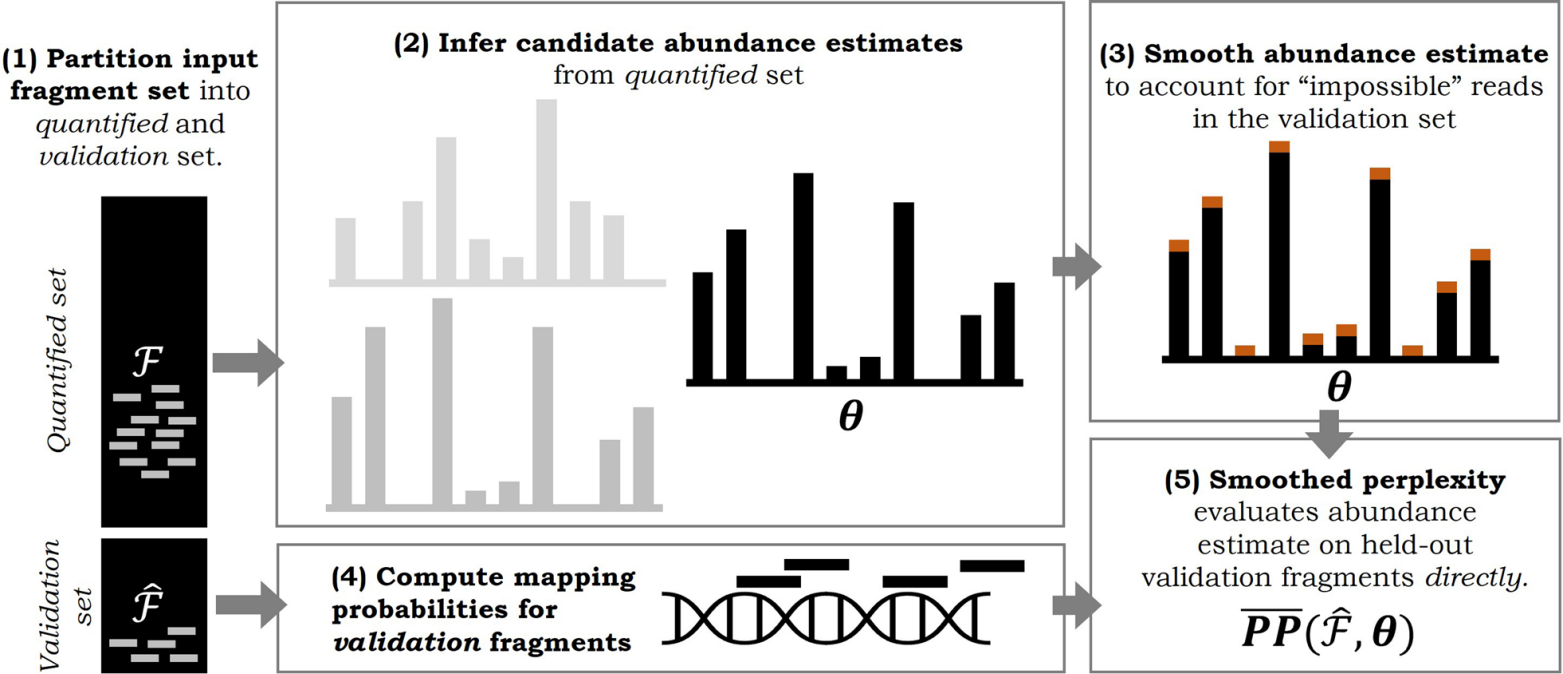
Overview of the *quantify-then-validate* approach using *smoothed perplexity* to evaluate the quality of abundance estimates directly on fragment sets in the absence of ground truth. (1) An input fragment set is first partitioned into a *quantified* and a *validation* set. (2) Abundance estimates for different candidate models (e.g. for explored hyperparameters as part of model selection) are inferred from the *quantified* fragment set only. (3) To account for “*impossible*” fragments and avoid shrinkage to unbounded perplexities, given abundance estimates are smoothed (see Sect. 3.2). (4) Mapping probabilities to the reference transcriptome are computed for fragments in the validation set. (5) *Smoothed perplexity* is computed given each input abundance estimate and the held-out validation fragment set to evaluate and perform model selection—the lower the perplexity, the better an abundance estimate describes the held-out set of validation fragments

We schematically illustrate how a smoothed perplexity measure, using the proposed quantify-then-validate protocol, can be computed to evaluate the quality of transcript abundance estimates in Fig. 2.

### Laplacian smoothed perplexity

We define *Laplacian smoothed perplexity* given abundance estimate $\mathbf {\theta }$ to be the perplexity evaluated with the smoothed distribution ${\widetilde{\mathcal {P}}}_\beta (t_i \mid \mathbf {\theta })$ in place of $\mathcal {P}(t_i \mid \mathbf {\theta })$. The Laplacian smoothing scheme smooths input abundance estimates by redistributing a small constant probability mass across the reference transcriptome. Let $\mathcal {P}(t_i \mid \mathbf {\theta }) = \eta _i$ and *M* be the number of transcripts in the reference. The smoothed distribution parameterized by $\beta$ is defined to be:

7 $$\begin{aligned} {\widetilde{\mathcal {P}}}_\beta (t_i \mid \mathbf {\theta }) = \frac{\eta _i + \beta }{1 + M\beta }. \end{aligned}$$

Laplacian smoothed perplexity is flexible and easy to implement but requires the user to *set* a value (preferably small e.g. $1\times 10^{-8}$) for the smoothing parameter $\beta$.^1^ At the cost of not being parameter-free, Laplacian smoothed perplexity allows the user to tune the degree to which impossible reads are penalized. The smaller the value of $\beta$, the smaller larger the penalty an estimate incurs for each validation fragment it calls impossible

### Good-Turing smoothed perplexity—an *adaptive*, “parameter-free” strategy

The major drawback of Laplacian smoothed perplexity is that it depends on a reasonable *a priori* selection of a value for the smoothing parameter $\beta$. One further concern is that Laplacian smoothed perplexity is not adaptive and does not account for the amount of evidence from which an input estimate is derived, i.e. the read-depth or the number of quantified reads in a sample. For a fixed value of $\beta$, the Laplacian smoothed perplexity smooths probabilities inferred from a million fragments equally as much as probabilities inferred from a trillion fragments. However, for the latter estimate that is inferred from much more data, it is more sensible to smooth and redistribute less probability mass.

For example while varying one of salmon’s hyperparameters, Laplacian smoothed perplexities suggest the existence of a locally optimal behavior when computed with a wide range of values for $\beta$ (see Fig. 16). However, the locally optimal behavior can no longer be observed if Laplacian smoothed perplexities are computed with $\beta =1\times 10^{-6}$.

A better, adaptive, smoothing strategy would directly estimate the probability of observing fragments from transcripts that are not expressed. Abstractly, the problem to be solved is to estimate the probabilities of observing unobserved events. Here, we turn to the Simple Good-Turing (SGT) method [30] that has been applied in a wide range of areas, including estimating the probabilities of unseen sequences in computational linguistics [30], as well as for the detection of empty droplets in droplet-based single-cell RNA sequencing protocols [31].

Below, we define the *Good-Turing smoothed perplexity* measure, where smoothed probabilities are derived from SGT smoothed fragment per-transcript counts.

Given frequencies over a population—i.e. the number of reads originating from each trancsript—the SGT method estimates:

1. the *total* probability mass that ought be assigned to unseen events—the “expression” of unexpressed transcripts, and
2. the appropriate adjustments for probabilities of observed events—the adjusted probabilities for expressed transcripts.

It is not immediately obvious how to implement SGT smoothing for the purpose of smoothing transcript abundance estimates. One issue is that the SGT estimator expects as input, integer-valued frequencies of observed events, while input abundance estimates for computing perplexity are real-valued estimated frequencies of per-transcript counts. For the purposes of smoothing and computing perplexity, we round the estimated number of fragments per-transcript, $c_i$, to the nearest integer and treat these as raw frequencies of events for SGT smoothing.

**Fig. 3:**
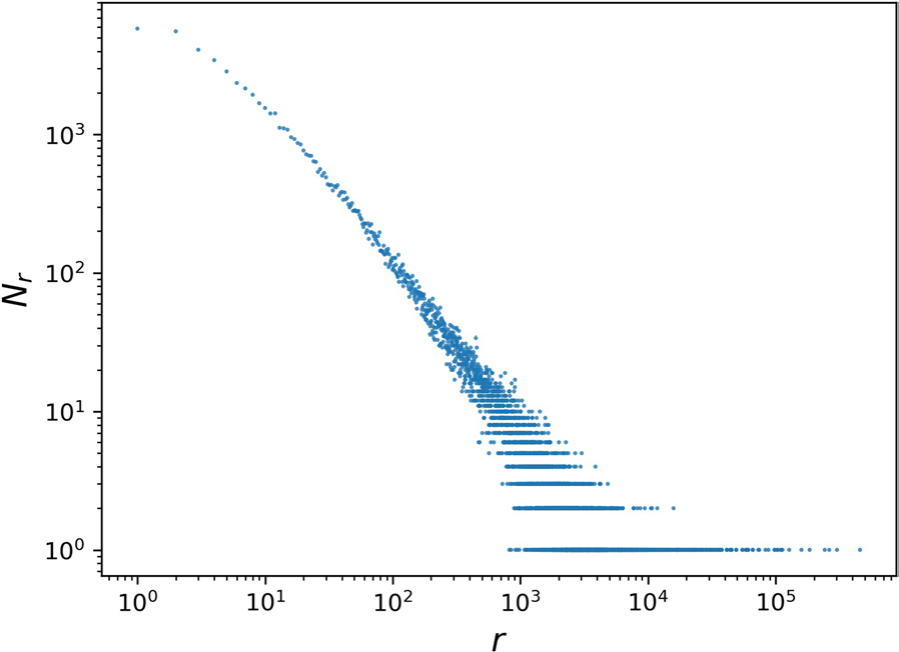
Frequencies-of-frequencies follow log-linear distribution for SEQC sample A1

The SGT method also requires that input frequencies-of-frequencies (i.e. the number of transcripts that have the same fragments per-transcript) to be log-linear. Empirically, we show in Fig. 3 that rounded input abundance estimates do, indeed, follow a log-linear distribution. The confirmed log-linear relationship demonstrates the rounding step to be a reasonable approximation.

The SGT method estimates the adjusted frequencies $r^\star$ for each event observed *r* times. These adjusted frequencies are then used to compute per-event (or per-transcript) probabilities. Let $c_i$ be the rounded number of fragments per-transcript $t_i$. Let the frequency of frequencies $n_r~=~|\{t_i~\mid ~c_i~=~r\}|$. And let there be ${\mathbf {n}}$ total reads. The SGT method computes and outputs,

1. the adjusted frequencies, $r^\star = (r+1) \frac{S(n_{(r+1)})}{S(n_r)}$;
2. and the total probability, $P_0 = \frac{n_1}{{\mathbf {n}}}$, for observing any transcript with $c_i = 0$.

Here, $S(n_r)$ computes a smoothed frequency of frequencies. Frequencies of frequencies $n_r$ have to be smoothed because $n_r$ for many large *r* are zero in observed data. The precise details for computing the smoothed $S(n_r)$ are described in [30]. In brief, SGT smooths $n_r$ by fitting a fitted log-linear function on *r* against $n_r$ and reading off values of $n_r$ for “large” *r*.

Good-Turing smoothed perplexity is perplexity computed with the smoothed per-transcript distribution ${\widetilde{\mathcal {P}}}(t_i\mid \mathbf {\theta })$ in place of $\mathcal {P}(t_i~\mid ~\mathbf {\theta })$. Here, the smoothed per-transcript distribution is derived from adjusted frequencies $r^\star$ and $P_0$.

For each “expressed” transcript $t_i$ with count, $c_i = r$, greater than zero, the SGT smoothed probability is proportional to the transcript’s adjusted frequency normalized by its effective length, with ${\widetilde{\mathcal {P}}}(t_i\mid \mathbf {\theta }) \propto r^\star /\tilde{\ell _i}$. The smoothed probabilities for expressed transcripts are normalized so that they sum to $(1-P_0)$.

For each “unexpressed” transcripts with $c_i = 0$, the SGT smoothed probability is proportional to the transcript’s effective length, and is derived from distributing the probability mass $P_0$ uniformly over the effective lengths of all unexpressed transcripts in the reference. Here, the smoothed per-transcript distribution is defined ${\widetilde{\mathcal {P}}}(t_i\mid \mathbf {\theta }) \propto P_0{\ell _i}$. The smoothed probabilities for unexpressed transcripts are normalized so that they sum to $P_0$.

For all following sections we shall use perplexity to mean Good-Turing smoothed perplexity unless stated otherwise.

### Model selection using perplexity in practice

Arguably, one of the most useful outcomes of being able to evaluate the quality of abundance estimates in the absence of ground truth is the ability to perform model selection for transcript abundance estimation in experimental data. For those familiar with train-then-test experimental protocols for model selection in machine learning or NLP, model selection for transcript abundance estimation *vis-a-vis* our proposed quantify-then-validate approach is analogous and identical in abstraction. However, since, to our knowledge, this work is the first to propose a quantify-then-validate approach for transcript abundance estimation, we shall briefly detail how perplexity ought to be used in practice.

Let us consider model selection via fivefold cross-validation using perplexity given some fragment set ${\mathbf {F}}$. First, ${\mathbf {F}}$ is randomly partitioned into five equal sized, mutually exclusive validation sets, $\{{\widehat{\mathcal {F}}}_1, \dots , {\widehat{\mathcal {F}}}_5\}$—and quantified sets are subsequently defined, $\mathcal {F}_i = {\mathbf {F}}-{\widehat{\mathcal {F}}}_i$. Now, suppose we desire to choose between *L* model configurations (e.g. from *L* hyperparameter settings). Then for each $\ell$-th candidate model, we produce a transcript abundance estimate from each *i*-th quantified set, $\mathbf {\theta }^{(\ell )}_i$. To select the best out of the *L* candidate models, one simply selects the model that minimizes the average perplexity over the five folds, $\frac{1}{5} \sum _i \overline{PP}({\widehat{\mathcal {F}}}_i, \mathbf {\theta }_i^{(\ell )})$.

One additional practical consideration should also be noted. Given *any* pair of quantification and validation sets $\mathcal {F}$ and ${\widehat{\mathcal {F}}}$, a validation fragment, ${\hat{f}}_j \in {\widehat{\mathcal {F}}}$, can be *necessarily impossible*. A necessarily impossible validation fragment is one that maps to a set of transcripts to which no fragments in the quantified set $\mathcal {F}$ also map. Such a fragment will always be called impossible given any abundance estimate deriving from the quantified set $\mathcal {F}$, since no fragments in $\mathcal {F}$ provide any evidence that transcripts to which ${\hat{f}}_j$ map are expressed.

It is of limited meaning to evaluate estimates with respect to necessarily impossible fragments. For the purposes of this work, we shall consider the penalization of an abundance estimate only with respect to impossible fragments that are recoverable—in other words, fragments that could be assigned non-zero probability given a better abundance estimate inferable from $\mathcal {F}$. As such, we remove necessarily impossible validation fragments from ${\widehat{\mathcal {F}}}$, given $\mathcal {F}$, prior to computing perplexity whenever fragment sets are partitioned into validation and quantified fragment sets.

### Data

#### Sequencing Quality Control (SEQC) project data

We downloaded Illumina HiSeq 2000 sequenced data consisting of 100+100 nucleotide paired-end reads from the Sequencing Quality Control (SEQC) project [20]. SEQC samples are labeled by four different conditions $\{A, B, C, D\}$, with condition *A* being Universal Human Reference RNA and *B* being Human Brain Reference RNA from the MAQC consortium [32], with additional spike-ins of synthetic RNA from the External RNA Control Consortium (ERCC) [33]. Conditions *C* and *D* are generated by mixing *A* and *B* in 3:1 and 1:3 ratios, respectively.

In this work, we analyze the first four replicates from each condition sequenced at the Beijing Genomics Institute (BGI)—one of three official SEQC sequencing centers. For each sample, we aggregate fragments sequenced by all lanes from the flowcell with the lexicographically smallest identifier.^2^ Quantitative PCR (qPCR) data of technical replicates for each sample in each condition are downloaded via the seqc BioConductor package.

#### Simulated lung transcript expression data

We simulated read-sets based on 10 sequenced healthy lung samples, with Sequence Read Archive accession number SRR1265{495-504} [34]. Transcript abundance estimates inferred by Salmon using the --useEM flag for each sample are used as ground truth abundances for read simulation (expressed in transcripts per million (TPM) and expected read-per-transcript counts). Then, transcript abundances in samples SRR1265{495-499}, for $10\%$ of transcripts expressed in at least one of the five samples, are artificially up or down regulated by a constant factor ($2.0\times$) to simulate differential transcript expression. We treat the resulting read-per-transcript counts as ground truth, and generate for each sample a fragments set of 100+100 nucleotide paired-end reads using Polyester at a uniform error rate of 0.001 with no sequence specific bias [35].

### Evaluation and experiments

The purpose of the experiments in this work are twofold. First, to establish the relationship and correspondence between perplexity and commonly used measures of goodness or accuracy in transcript abundance estimation. And second, to demonstrate how model and hyperparameter selection can be performed using perplexity. In particular, we perform and evaluate hyperparameter selection for salmon with respect to the prior size in the variational Bayesian expectation maximization (VBEM) model used for inference [15]. The user-selected prior size for the VBEM model in salmon encodes the prior belief in the number of reads-per-transcript expected for any inferred abundance estimate. This hyperparameter controls salmon’s preference for inferring sparse or smooth estimates—the smaller the prior size, the sparser an estimate salmon will prefer. As discussed above, prior studies on Bayesian models have not necessarily agreed on how sparse or smooth a good estimate ought to be [11, 12]—the experiments in this work aim to provide a quantitative framework to settle this disagreement.

We perform all experiments according to the proposed quantify-then-validate procedure and report results with respect to various metrics over a fivefold cross-validation protocol. We use the Ensembl human reference transcriptome GRCh37 (release 100) for all abundance estimation and analysis [36].

#### Evaluation versus parallel SEQC qPCR measurements

We analyze the relationship between perplexity and accurate abundance estimation in experimental data from the SEQC consortium. In SEQC data, we evaluate accuracy of abundances estimated by salmon by comparing estimates to qPCR gene expression data on biological replicates, a coarse proxy to ground truth. We evaluate the Spearman correlation between gene expressions of qPCR probed genes in SEQC replicates versus the corresponding abundance estimates. Gene expression from estimated transcript expression is aggregated via txImport [6] with transcript-to-gene annotations from EnsDb.Hsapiens.v86 [37]. From gene expression data, Ensembl genes are mapped to corresponding Entrez IDs via biomaRt [38], and 897 genes are found to have a corresponding qPCR measurement in downloaded SEQC data. Expressions for genes with repeated entries in SEQC qPCR data are averaged.

#### Evaluation versus ground truth on simulated data

In simulated data, since ground truth abundances are available, we compare estimated TPMs (computed by salmon) against ground truth TPMs under two metrics.

**Fig. 4:**
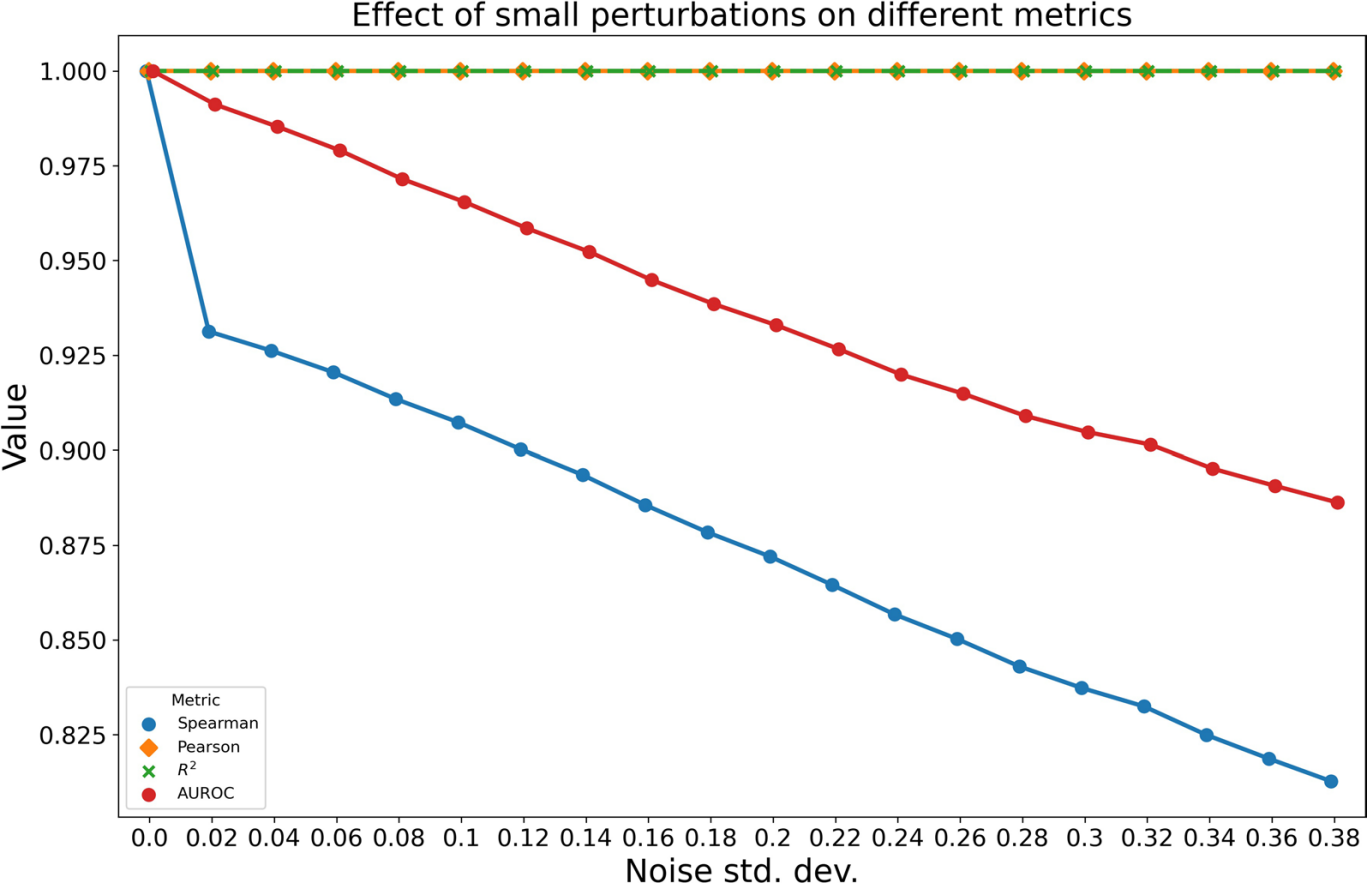
Spearman correlation, Pearson correlation and $R^2$ with respect to all transcripts in the reference, and AUROC for recalling ground truth unexpressed transcripts, with respect to added normally distributed noise with varying standard deviations. Plotted lines for Pearson correlation and $R^2$ overlap

First, we consider the Spearman correlation with respect to known expressed transcripts (i.e. transcripts with non-zero expression in ground truth abundances). We choose to evaluate Spearman correlation with respect to ground truth non-zero TPMs because of the presence of many unexpressed transcripts in the ground truth, meaning a high number of values tied at rank zero. Here, small deviations from zeros can lead to large changes in rank, leading to non-trivial differences in the resulting Spearman correlation metric. We demonstrate this phenomenon with respect to the ground truth abundance of a simulated sample (SRR1265495) with a mean TPM of 5.98, in which 49% of transcripts are unexpressed (82,358 / 167,268). We report the change in Pearson correlation, $R^2$ score, and Spearman correlation of ground truth TPMs versus ground truth TPMs perturbed with normally distributed noise at varying standard deviations. As we can see from Fig. 4, even small perturbations cause non-trivial changes in Spearman rank correlation, while changes in Pearson correlation are entirely imperceptible. The Pearson correlation, however, suffers from the well known problem that, in long-tailed distributions spanning a large dynamic range, like those commonly observed for transcript abundances, the Pearson correlation is largely dominated by the most abundant transcripts.

Second, we complement measuring Spearman correlation of non-zero ground truth TPMs with reporting the area under receiver operating characteristic (AUROC) for recalling ground truth zeros based on estimated abundances. While the measurement of Spearman correlation on the truly expressed transcripts is robust to small changes in predicted abundance near zero, it fails to account for false positive predictions even if they are of non-trivial abundance. The complementary metric of the AUROC for recalling ground truth zeros complements that metric, since it is affected by false positive predictions.

#### Differential expression analysis on simulated data

We perform transcript level differential expression analysis and analyze the recall of known differentially expressed transcripts in simulated lung tissue data (see 3.6.2). We perform differential expression analysis at the trancript level using swish [39] using 20 inferential replicates from salmon. We modified salmon to ensure that prior sizes supplied via the --vbPrior flag are propagated to the Gibbs sampling algorithm. We plot receiver operating characteristic (ROC) curves and report the mean AUROC for predicting differentially expressed transcripts over multiple folds. We assign $P = 1$ to transcripts for which swish does not assign adjusted P-values.

#### Evaluation of eXpress abundance estimates

We measure the change in perplexities of abundance estimates inferred by eXpress (version 1.5.1) when running 0, 1, and 2 additional rounds of the online expectation maximization (EM) optimization step. We specify the number of additional online EM steps using the –additional-online parameter. We provide to eXpress alignments to the human transcriptome computed by bowtie2 [40] using the parameters recommended by eXpress with: -a -X 600 –rdg 6,5 –rfg 6,5 –score-min L,-.6,-.4 –no-discordant –no-mixed.

To compute perplexity, we use the transcript effective lengths computed by eXpress for each transcript inferred to be expressed. For each transcript inferred to be unexpressed, we use transcript lengths in place of effective lengths, since eXpress sets the effective length for these transcripts to zero. We take effective counts computed by eXpress to be expected fragment per-transcript counts.

### Implementation

We implement perplexity in Rust and provide snakemake [41] workflows to (a) set up quantify-validate splits of fragment-sets for K-fold cross-validation, and (b) compute perplexities of salmon abundance estimates with respect to validation fragment sets at: github.com/COMBINE-lab/perplexity. Approximate per-fragment probabilities (Eq. 6) are computed by running salmon with options –skipQuant and –dumpEq. Code to reproduce the experiments and figures for this work is available at github.com/COMBINE-lab/perplexity-paper.

## Results

### Low perplexity implies accurate abundance estimates in experimental SEQC data

**Fig. 5:**
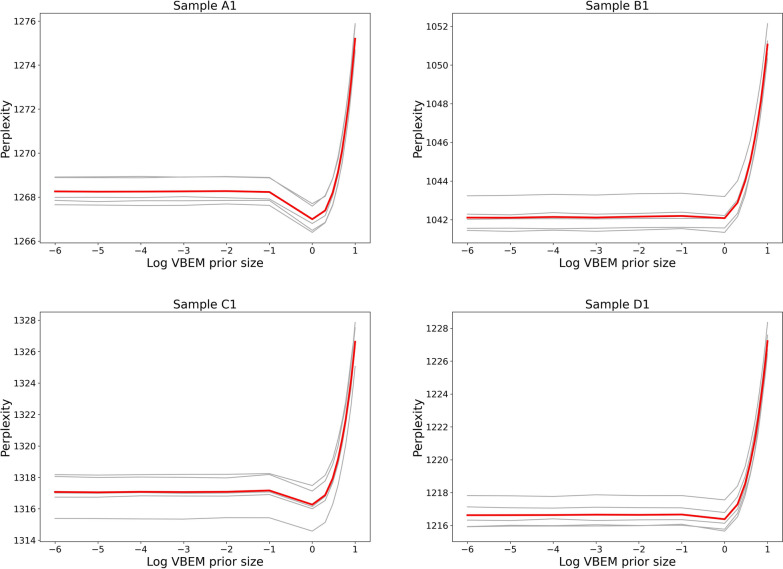
Perplexity plots for SEQC samples. Plots show perplexity versus VBEM reads-per-transcript prior size for SEQC samples—plots only for the first replicate of samples from conditions *A*-*D* are shown. Perplexity plots for other replicates are consistent within condition and are included in Appendix. Mean perplexities across five folds are plotted in red, and perplexities for each fold are plotted in gray

**Fig. 6:**
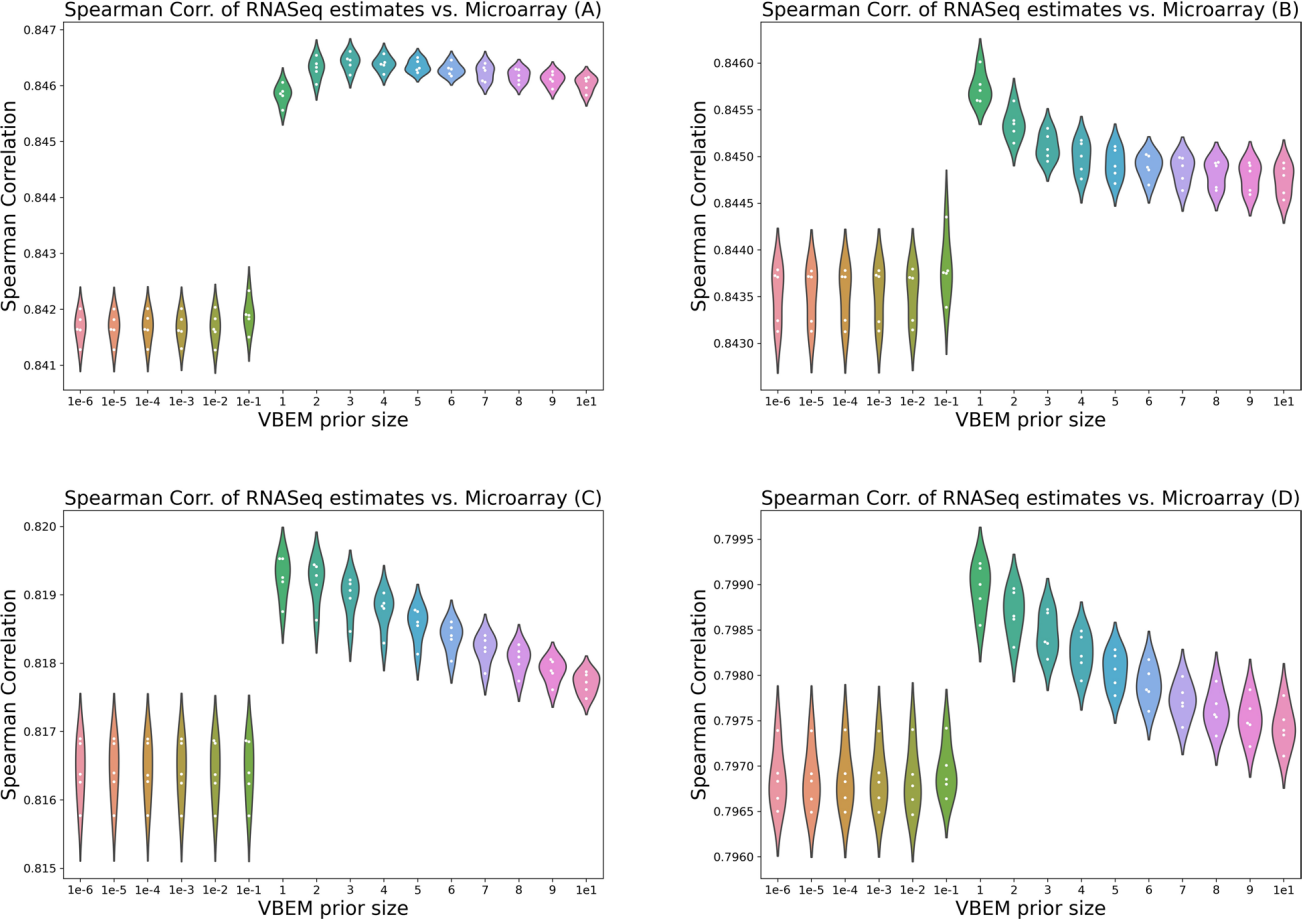
Spearman correlation of abundance estimates at various VBEM reads-per-transcript prior sizes, versus parallel qPCR microarray gene-expression measurements conditions *A*-*D*. Each point in above plots indicate the mean correlation across replicates for a given fold

In experimental data from the Sequencing Quality Control (SEQC) project [20], we demonstrate that perplexity can be used to perform parameter selection and select the salmon VBEM prior size that leads to the most accurate transcript abundance estimates. We note that perplexity plots for replicates are similar within conditions *A*-*D*, and thus include only plots for the first replicate in each condition in the main text. For completeness, plots for all samples are presented in Appendix (Figs. 10, 11, 12,  13).

Empirically, perplexity is well-behaved over all samples in the experimental data. As shown in Figs. 5 and 6, plots of perplexity against VBEM prior size and Spearman correlation against VBEM prior size both display an empirically convex shape minimized at the same VBEM prior size. This suggests that minimizing perplexity is, at least, locally optimal with respect to the set of explored hyperparameters.

Furthermore, for almost all samples, perplexity is minimized where correlation with qPCR measurements is maximized. For all replicates in conditions $\{B, C, D\}$, estimates that minimize perplexity with respect to held-out validation fragments achieve the best correlation with qPCR measured gene expression. For replicates in these conditions, abundances inferred using a prior size of 1 read-per-transcript resulted in estimates with the lowest perplexity. In replicates from condition *A*, estimates with lowest perplexity are significantly better than estimates at default hyperparameter settings (0.01 reads-per-transcript).

Perhaps surprisingly, both perplexity and correlation against qPCR measurements prefer a reads-per-transcript prior size that is larger than the 0.01 reads-per-transcript that is the current default for the salmon VBEM model. Selecting a larger per-transcript prior for transcript abundance estimation with salmon results in estimates that are more smooth. Compared to a sparser estimate, a smoother abundance estimate likely calls fewer validation time fragments impossible. Here, the number reads an estimate calls of impossible is symptomatic of two kinds of inferential errors—that some transcripts are incorrectly inferred to be unexpressed, and that other transcripts are assigned inaccurate inferred expression.

Without perplexity, it would be difficult to determine empirically, or *a-priori*, that a VBEM prior size of 1 is an optimal parameter setting since no comparison to ground-truth is possible. To the best of our knowledge, this experiment is the first to carry out both an effective and ubiquitously applicable quantitative strategy to perform model selection in the context of transcript abundance estimation on experimental data in the absence of ground truth.

### Perplexity versus ground truth, and differential expression analysis in simulated data

**Fig. 7:**
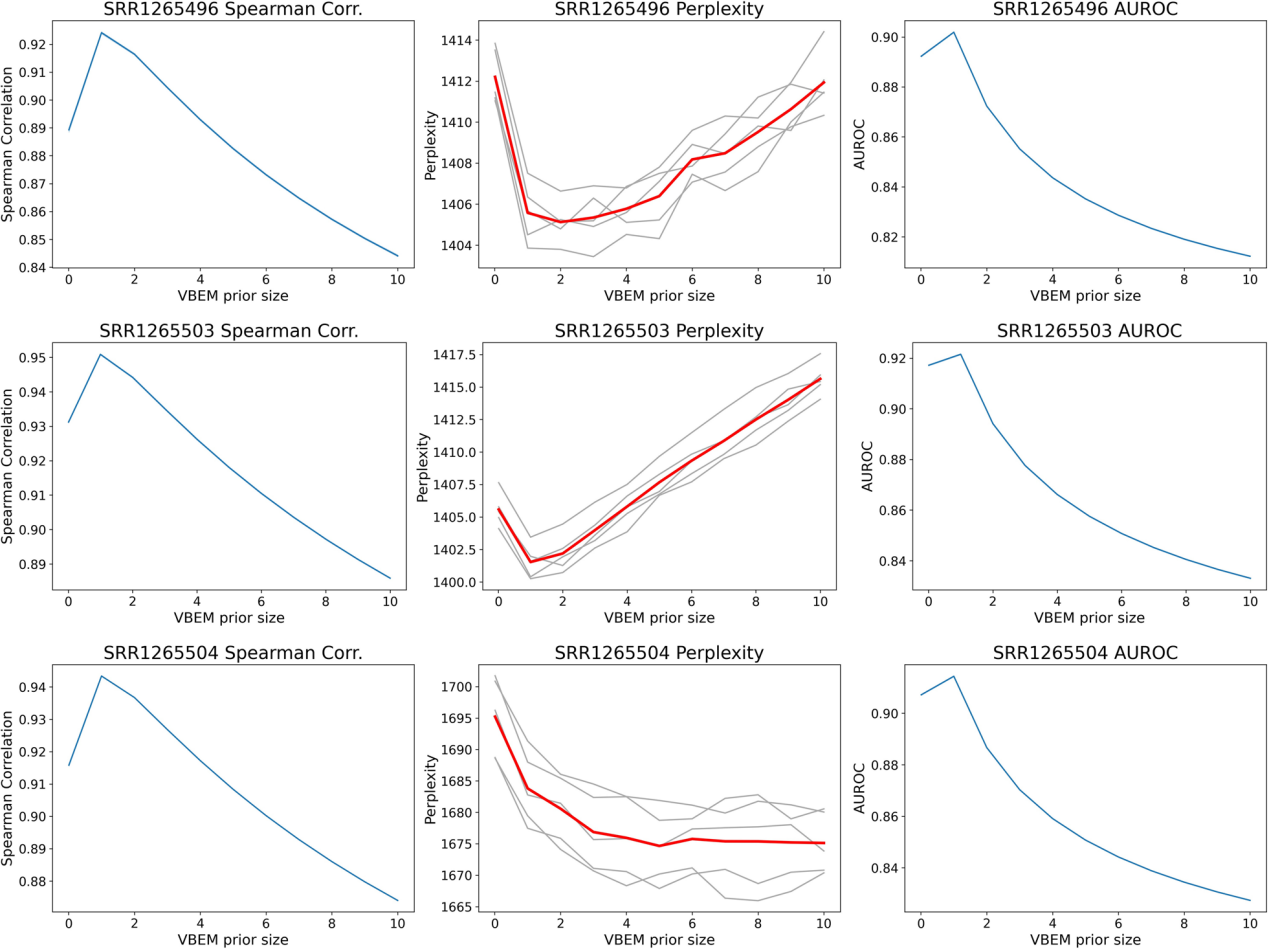
Quality of transcript abundance estimates as a function of VBEM per-nucleotide prior size for samples SRR1265{496,503,504}. (Left column) Spearman Correlation with respect ground truth expressed transcripts. (Middle column) Perplexity of abundance estimates; perplexities per-fold indicated in gray and mean perplexities in red. (Right column) AUROC for retrieving ground truth unexpressed transcripts. Leftmost plotted points for all plots use default salmon VBEM prior size of 0.01 reads-per-transcript

**Fig. 8:**
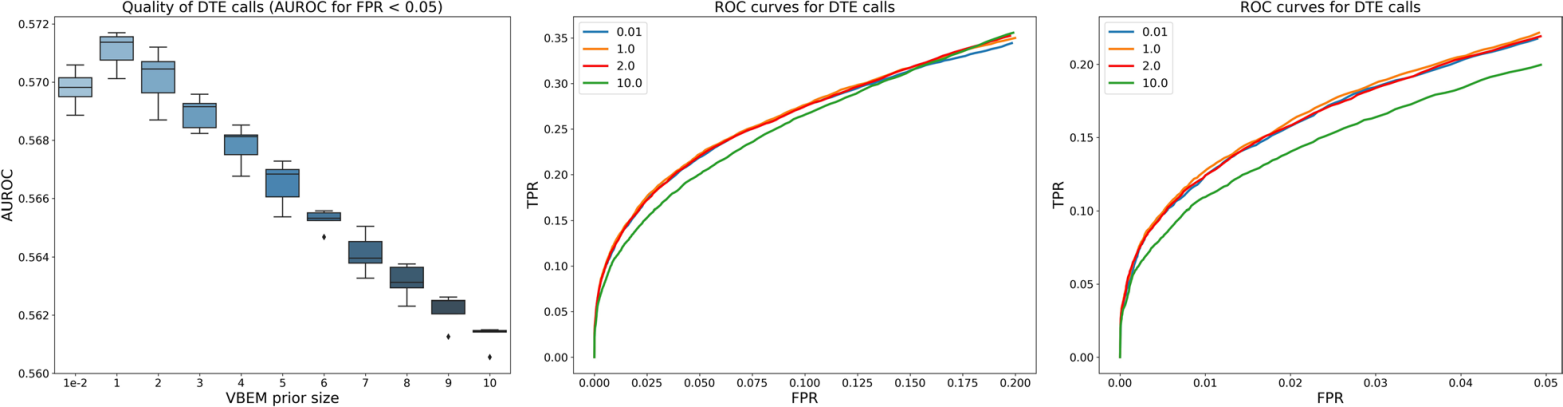
Accuracy of differential expression analysis with respect to experiment-wide selection of VBEM per-nucleotide prior size. (Left) AUROC with respect to DTE calls at real FPRs up to 0.05. (Middle) ROC curve up to FPR = 0.20. (Right) ROC curve up to FPR = 0.05. To reduce visual clutter, only the ROC curves some representative VBEM prior size settings are plotted

In simulated data, the relationship between perplexity and measurements against ground truth, though well-behaved, is admittedly less direct. In short, under the implemented experimental framework, minimizing perplexity does not always find the best performing estimates. Across all 10 samples, perplexity prefers abundance estimates that are smoother than estimates that are most accurate when compared to ground truth. For brevity, we include in the main text perplexity plots of three samples (SRR1265{496,503,504}) that are representative of three main modalities of perplexity plot behaviors (Fig. 7). For completeness, and plots for all samples are presented in Appendix (Figs. 14 and 15).

In all but two samples (SRR1265{497,504}), perplexity plots display a empirically convex shape with a local minima close to the optimal VBEM prior size (1 read-per-transcript). For example, for sample SRR1265503, perplexity is minimized at a VBEM prior setting of 1 reads-per-transcript, *exactly* the best performing hyperparameter setting with respect to Spearman correlation (Fig. 7; middle). And for sample SRR1265496, we can see that perplexity prefers VBEM prior setting in a wide local minima ranging from 1 to 3 reads-per-transcript (Fig. 7; top). Sample SRR1265504 is one sample for which a local minimal perplexity cannot be identified with respect to the range of hyperparameters scanned (Fig. 7; bottom). However, the perplexity plot for SRR1265504 displays a knee-like behavior which suggests that after a certain VBEM prior size, larger VBEM prior sizes are no longer preferred—which is consistent across all perplexity plots and comparisons to ground truth.

These experiments in simulated data suggest that, perhaps, perplexity remains an imperfect tool. Nonetheless, these observations do offer insights about how perplexity ought to be used in practice. First, perplexities may prefer abundance estimations smoother than ideal. In particular, when perplexities for two VBEM prior settings are close, or when perplexities are roughly minimized for a range of values, one ought to select the model that outputs the sparsest estimates. Second, careful (albeit qualitative) inspection of perplexity plots can be used to select an optimal hyperparameter setting experiment-wide. For example, inspection of perplexity plots (Figs. 14 and 15) over all samples show either knee-like behaviors beginning at, or local minimas centered close to a VBEM prior size of 1 reads-per-transcript—the best hyperparameter setting.

Notably, the results also show that perplexity can simply be used to quantitatively reject poor abundance estimates (or the hyperparameters that generate them). Although the significance of this property may be overlooked at first, perplexity is to our knowledge the only metric that can do so when ground truth is not available.

We also analyze the accuracy of differential transcript expression (DTE) analysis of estimates with the same VBEM prior size experiment-wide. We report AUROC of DTE calls up to a nominally useful maximum false discovery rate (FDR) of 0.05 (Fig. 8). Not surprisingly, AUROC of DTE calls mirror the shape of Spearman correlations of estimates inferred from different VBEM prior sizes. Again, for each individual sample, minimizing perplexities may not always select the best hyperparameter setting. But, experiment-wide, perplexity plots do begin to exhibit minima or knee-like behaviors at VBEM prior size of 1 reads-per-transcript—the best performing hyperparameter setting with regard to DTE (Fig. 8).

### Perplexity measures improved accuracy due to additional eXpress online optimization rounds

Crucially, perplexity can be used to evaluate the performance of arbitrary abundance estimators that output per-transcript probabilities $\mathcal {P}(t_i | \theta )$. This is because, perplexity is computed from decoupled per-transcript terms $\mathcal {P}(t_i \mid \mathbf {\theta })$ from abundance estimates inferred only from the quantification fragment set, and per-fragment terms $\mathcal {P}(f_j \mid t_i)$ from mapping probabilities calculated only from the the validation fragment set. The comparison of different models simply requires agreement on per-fragment probabilities $\mathcal {P}({\hat{f}}_j | t_i)$ for all fragments in the validation set. Per-fragment probabilities $\mathcal {P}({\hat{f}}_j | t_i)$ can simply be computed from any tool that makes these available (e.g. salmon).

**Fig. 9:**
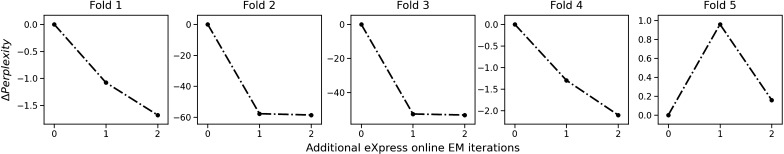
Change in perplexity from additional eXpress online expectation-maximization (EM) rounds. Reduction in perplexity indicates improved quality of estimated abundances after each online EM round

Thus, perplexity can be especially useful for investigating and verifying specific behaviors of different abundance estimation algorithms. To demonstrate this, we explore how perplexities can be calculated to investigate the improvement due to additional online optimization rounds when running eXpress [42]. eXpress uses a streaming optimization algorithm—online expectation-maximization (EM)—to quantify transcript abundance from the alignments of RNA-seq reads. Theoretically and empirically, additional rounds of the online-EM step is known to improve accuracy. Without perplexity, this behavior can only be verified when parallel measurements in experimental data are available(e.g. qPCR on biological replicates). With perplexity, this behavior can be verified from a sample’s fragment-set directly. According to perplexities shown in Fig. 9, running eXpress using one or two additional online EM rounds results in improved abundance estimates in four out of five folds. In this case, the perplexity results concord with the expectation that additional rounds of the online-EM step improves convergence and lead to improved estimates of transcript abundance.

When running an inference algorithm, a user can go beyond simply verifying that an abundance estimation model converges on input, quantified fragments. With perplexity, a user can now verify that said model generalizes, and is accurate with respect to held-out, validation fragments drawn *exactly* from the “true” latent distribution.

## Conclusions

In this work, we derive the smoothed perplexity metric, which, to our knowledge, is the first metric that enables the evaluation of the quality of transcript abundance estimates in the absence of ground truth.

In experimental data from the Sequencing Quality Control (SEQC) project [20], we show that the most accurate abundance estimates consistently have the lowest perplexity (lower is better) and demonstrate how quantitative model selection can be performed on input fragment sets directly and in the absence of ground truth. In simulated samples, we demonstrate a looser, but still useful, relationship between perplexity and measurements against ground truth. One possible explanation for the more erratic behavior and noisier perplexity plots for our simulated samples is due to these samples consisting of many fewer fragments than SEQC samples. On average, the simulated samples contain 17,410,732 fragments on average while the SEQC samples average 47,589,281 fragments.

Although we only demonstrate model selection with respect to only one hyperparameter (the VBEM prior size) in salmon using perplexity, model selection for other hyperparameters are possible with simple changes to the experimental protocols implemented here. For example, perplexity evaluated to choose the number of bins for the range-factorized likelihood approximation, or select between VBEM and EM models and optimization algorithms in salmon.

Notably, perplexity may be useful for investigating and comparing different abundance estimation models. In a proof-of-concept style experiment running eXpress [42], we demonstrate perplexity can be computed to verify theoretically predicted behavior. In doing so, we theoretically and empirically demonstrate that perplexity can be computed for *almost any* transcript abundance estimation model.

In future work, perplexity can perhaps be adapted and applied to other problem settings in bioinformatics where probabilistic models infer abundances. For example, perplexity may be useful in metagenomics where model selection (i.e. choosing confidence cutoffs for taxa identification, or selecting candidate reference genomes) can have a large effect on the quality of inferred abundances [43].

In sum, this work demonstrates that evaluation of transcript abundance estimates in the absence of ground truth is indeed possible. Perplexity is an example of a promising new direction in which estimated abundances can be evaluated and validated *directly* on input fragments themselves. This may prove fruitful not only for the re-analysis of previously published data where ground truth was absent, but also for current and future experimental settings where parallel experimental measurements complementary to RNASeq are too expensive or cumbersome to obtain.

## Figures and Tables

**Fig. 10:**
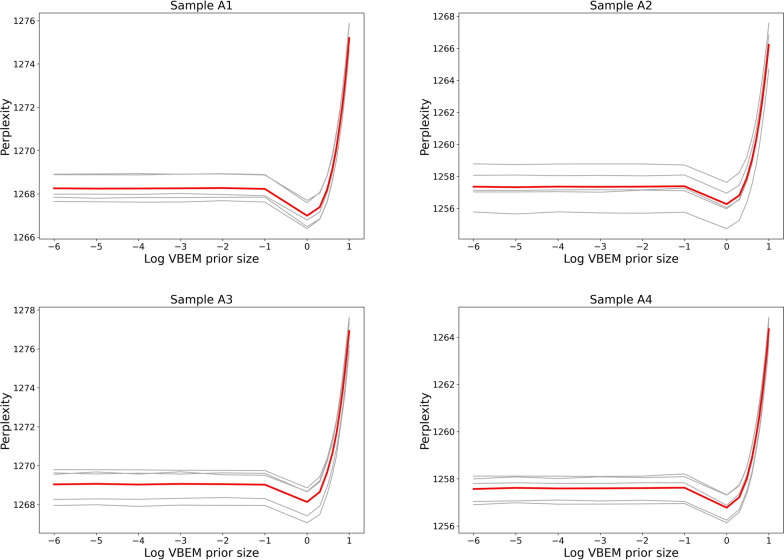
Perplexity plots for SEQC A samples. Plots show perplexity versus VBEM reads-per-transcript prior size for SEQC samples. Mean perplexities across five folds are plotted in red, and gray perplexities for each fold are plotted are plotted in gray

**Fig. 11:**
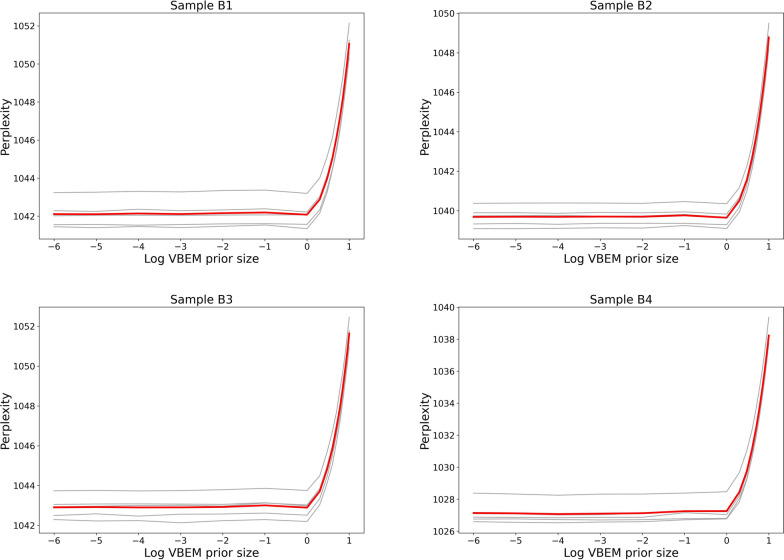
Perplexity plots for SEQC B samples. Plots show perplexity versus VBEM reads-per-transcript prior size for SEQC samples. Mean perplexities across five folds are plotted in red, and gray perplexities for each fold are plotted are plotted in gray

**Fig. 12:**
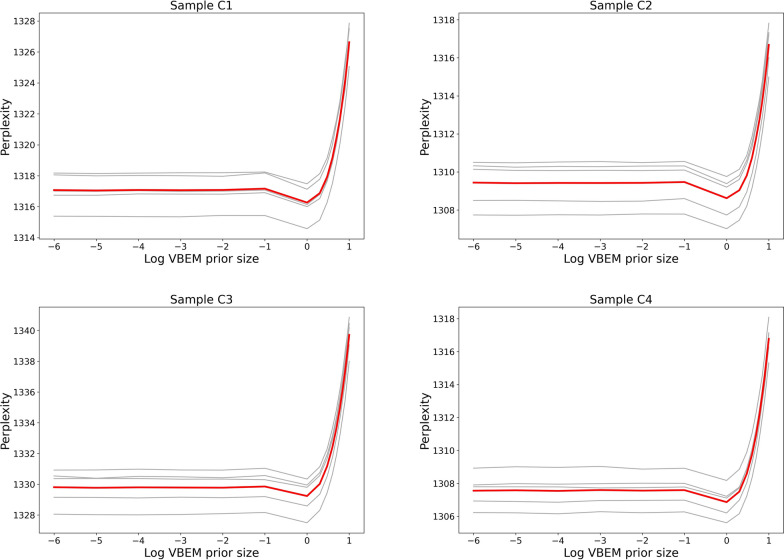
Perplexity plots for SEQC C samples. Plots show perplexity versus VBEM reads-per-transcript prior size for SEQC samples. Mean perplexities across five folds are plotted in red, and gray perplexities for each fold are plotted are plotted in gray

**Fig. 13:**
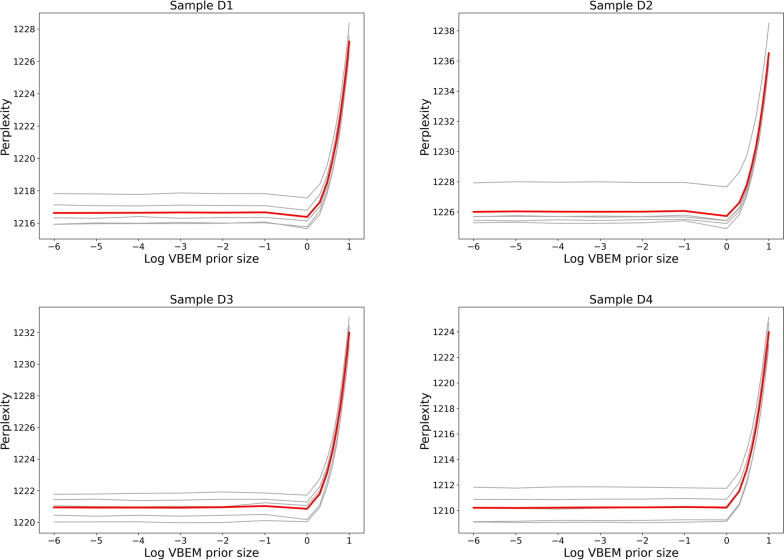
Perplexity plots for SEQC D samples. Plots show perplexity versus VBEM reads-per-transcript prior size for SEQC samples. Mean perplexities across five folds are plotted in red, and gray perplexities for each fold are plotted are plotted in gray

**Fig. 14:**
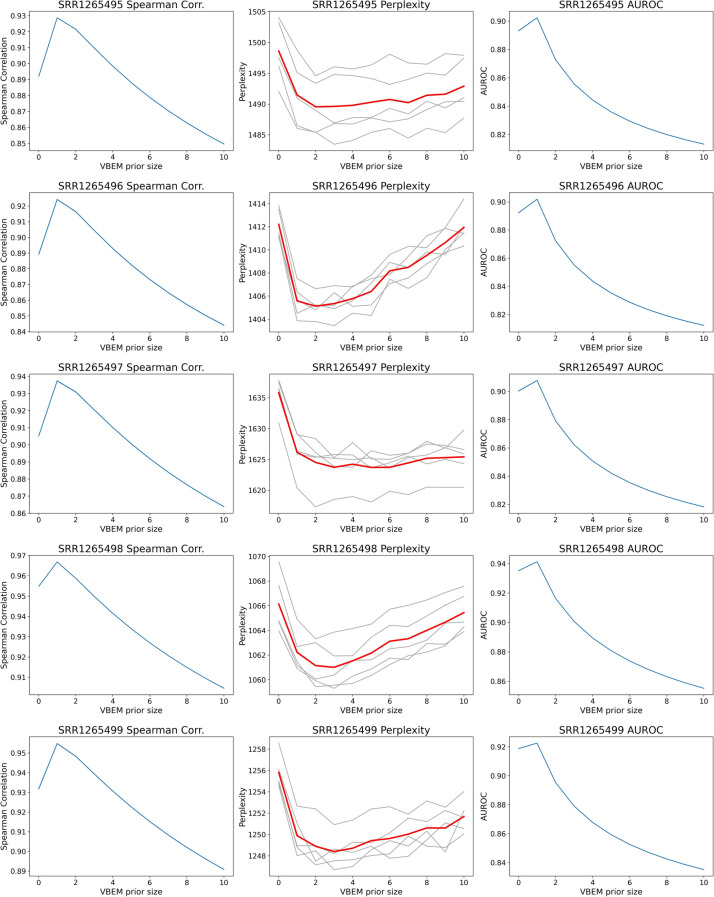
Quality of transcript abundance estimates as a function of VBEM per-nucleotide prior size for samples SRR1265{495-499}. (Left column) Spearman Correlation with respect ground truth expressed transcripts. (Middle column) Perplexity of abundance estimates; perplexities per-fold indicated in gray and mean perplexities in red. (Right column) AUROC for retrieving ground truth unexpressed transcripts. Leftmost plotted points for all plots use default salmon VBEM prior size of 0.01 reads-per-transcript

**Fig. 15:**
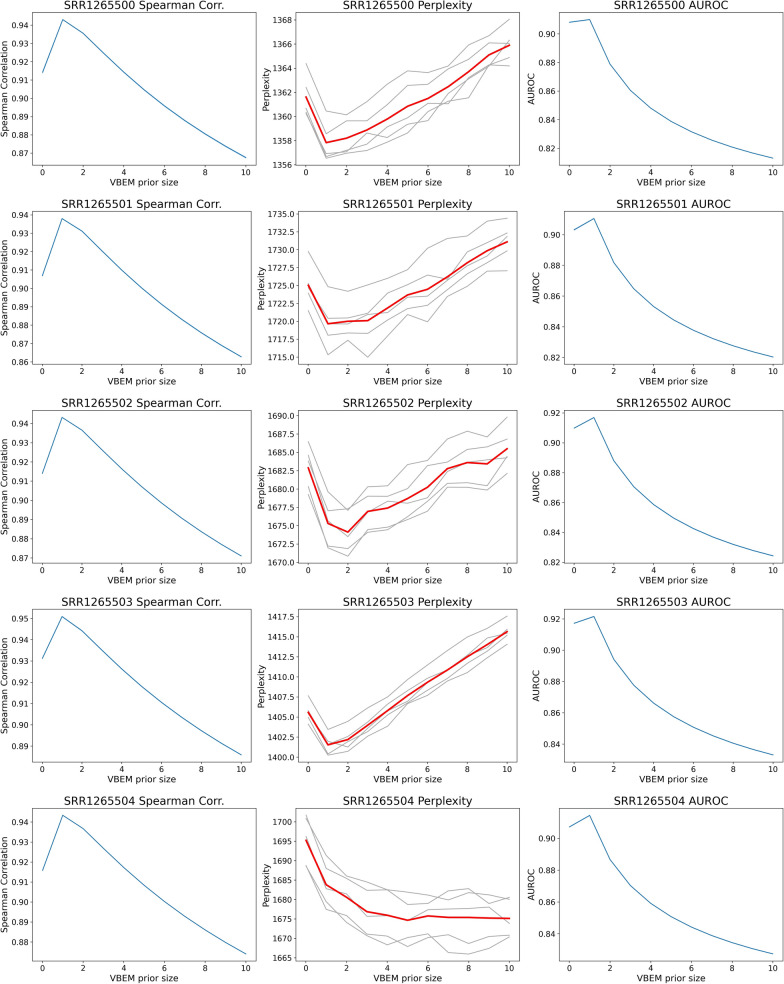
Quality of transcript abundance estimates as a function of VBEM per-nucleotide prior size for samples SRR1265{500-504}

**Fig. 16:**
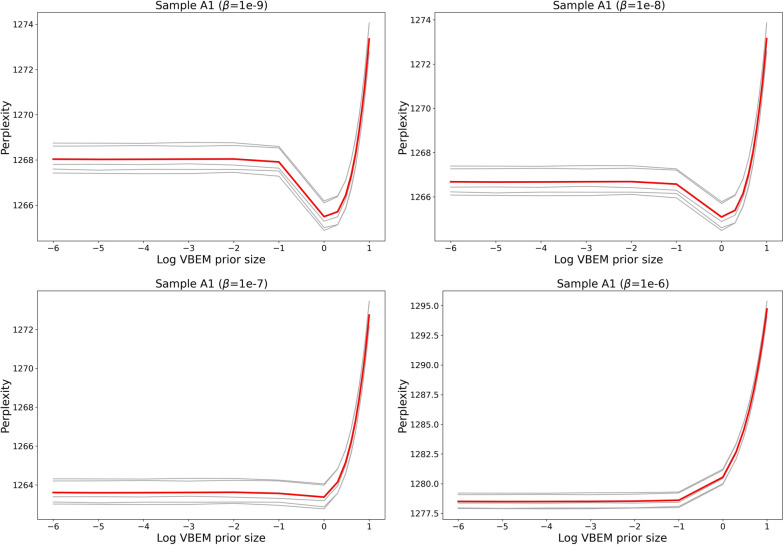
Perplexity plots for SEQC sample *A*1 at different smoothing parameter settings. Plots show perplexity versus VBEM reads-per-transcript prior size for SEQC samples. Mean perplexities across five folds are plotted in red, and gray perplexities for each fold are plotted are plotted in gray

## Appendix

Additional figures are included as Figs. 10, 11, 12, 13, 14, 15, 16.

## Footnotes

1 This is equivalent to adding, for each transcript $t_i$ in the reference, $\beta \cdot \sum _j^M c_j / {\tilde{\ell }}_j$ reads-per-nucleotide to the expected fragments per-transcript counts $c_i$ then re-normalizing to obtain TPMs, given effective transcript lengths ${\tilde{\ell }}_i$ (as defined in salmon [15]).

2 Scripts to download and aggregate SEQC data are available at github.com/thejasonfan/SEQC-data.
